# Supplementary material for: MetaRibo-Seq measures translation in microbiomes
Source: Nat Commun. 2020 Jun 29;11:3268. doi: 10.1038/s41467-020-17081-z (PMC7324362; doi:10.1038/s41467-020-17081-z)
Supplement: Supplementary file 10 — Supplementary Data 7 [file 41467_2020_17081_MOESM10_ESM.zip › File2/Confidence_High_Taxonomy/202038_out.krona.html]

Javascript must be enabled to view this page.

members
magnitude
magnitudeUnassigned
count
unassigned
taxon
rank

202038\_out

46

46
2
superkingdom

46
1239
phylum

class
46
186801

order
46
186802

family
186803
1

genus
841
1

1
166486
species

SRS063040\_contig\_number\_36319

45
186806
family


SRS148424\_contig\_number\_contig-100\_5250.105081
1730
45
genus
1


SRS016203\_contig\_number\_4125SRS018351\_contig\_number\_1331SRS020233\_contig\_number\_28132SRS049896\_contig\_number\_33431SRS075984\_contig\_number\_18449SRS1041129\_contig\_number\_contig-100\_1342.40808SRS1055043\_contig\_number\_21720SRS140492\_contig\_number\_33552SRS147271\_contig\_number\_4153SRS147977\_contig\_number\_12848SRS148721\_contig\_number\_51266
species
142586
11

species

SRS013951\_contig\_number\_30649SRS015782\_contig\_number\_42235SRS017307\_contig\_number\_17014SRS023526\_contig\_number\_contig-100\_27365.111216SRS023583\_contig\_number\_contig-100\_26727.26727SRS024388\_contig\_number\_contig-100\_2375.77431SRS024492\_contig\_number\_12256SRS043701\_contig\_number\_contig-100\_29265.29265SRS045645\_contig\_number\_26053SRS046717\_contig\_number\_7861SRS047044\_contig\_number\_3080SRS050925\_contig\_number\_contig-100\_33448.33448SRS051031\_contig\_number\_2804SRS053335\_contig\_number\_672SRS054905\_contig\_number\_12636SRS077335\_contig\_number\_9083SRS077641\_contig\_number\_21501SRS098644\_contig\_number\_15065SRS1041145\_contig\_number\_34055SRS1055022\_contig\_number\_12238SRS1055067\_contig\_number\_10192SRS143342\_contig\_number\_13427SRS143598\_contig\_number\_contig-100\_946.141211SRS147272\_contig\_number\_1022SRS148196\_contig\_number\_26845SRS148874\_contig\_number\_11968SRS893285\_contig\_number\_3954SRS893358\_contig\_number\_4326SRS893373\_contig\_number\_contig-100\_13442.54297
39485
29


SRS097958\_contig\_number\_contig-100\_9668.9669SRS144362\_contig\_number\_contig-100\_2581.2581SRS149181\_contig\_number\_contig-100\_1127.152601SRS893300\_contig\_number\_928
species
4
1263077
